# A New Method for Artificial Respiration in Asphyxia Neonatorum

**Published:** 1893-02

**Authors:** 


A NEW METHOD FOR ARTIFICIAL RESPIRATION IN ASPHYXIA NEONATORUM.—Forest (*MedicalRecord,* April 9, 1892), describes a new method of artificial respiration in asphyxiated children after labor.

The asphyxiated child is first laid for a moment upon its face, allowing the head to be a little lower than the pelvis. A light pressure is then exerted upon the back in order that the aspirated fluids may find exit from the mouth and trachea. It is then to be placed, in sitting position, in a bath of warm water up to the breast. The operator seizes with the left hand the wrists, the child's palms looking outward, while with his right hand he supports the shoulders, holding the head backward by the thumb and fingers. The child's arms are elevated and drawn a little backwards, during which movement they are rotated a little outward. The muscles between the shoulders and ribs are stretched and the ribs are elevated. The abdominal muscles are relaxed through the sitting position and do not oppose the rib elevation, as in the methods of Sylvester and Schultze. During this maneuvre the head of the child falls further backward, thereby closing the esophagus by pressure between the spinal vertebre and the laryngeal and thoracic cartilages, so that no air can enter. The tongue and epiglottis are at the same time drawn better forward than it were possible to do with forceps. In order to bring air into the lungs through the now freely opened air passages, the operator bends forward and blows, with his mouth upon the mouth of the child. The air now distends the lungs without the necessity of the introduction of the catheter into the larynx. For the expiration, the child's body is bent forward, whereby the abdominal organs are pressed upon the diaphragm, at the same time the left hand of the operator is lightly pressed upon the breast which is compressed between his two hands. The movements are then to be repeated.—*Ex.*

